# Hydrophilic Biocompatible Fluorescent Organic Nanoparticles as Nanocarriers for Biosourced Photosensitizers for Photodynamic Therapy

**DOI:** 10.3390/nano14020216

**Published:** 2024-01-19

**Authors:** Isabelle Sasaki, Frédérique Brégier, Guillaume Chemin, Jonathan Daniel, Justine Couvez, Rayan Chkair, Michel Vaultier, Vincent Sol, Mireille Blanchard-Desce

**Affiliations:** 1 Institut des Sciences Moléculaires (ISM, UMR5255), University of Bordeaux, Centre National de la Recherche Scientifique, Institut Polytechnique de Bordeaux, Bat A12, 351 Cours de la Libération, 33405 Talence, Francejustine.couvez@u-bordeaux.fr (J.C.);; 2 Laboratoire des Agroressources, Biomolécules et Chimie pour l’Innovation en Santé (LABCiS, UR22722), University of Limoges, 87000 Limoges, France; frederique.bregier@unilim.fr (F.B.); guillaume.chemin@unilim.fr (G.C.);

**Keywords:** PDT, chlorins, purpurin Pp18, nanocarrier, fluorescence, cell viability, colorectal cancer cell lines

## Abstract

Most photosensitizers of interest for photodynamic therapy—especially porphyrinoids and chlorins—are hydrophobic. To circumvent this difficulty, the use of nanocarriers is an attractive strategy. In this perspective, we have developed highly water-soluble and biocompatible fluorescent organic nanoparticles (FONPs) made from citric acid and diethyltriamine which are then activated by ethlynene diamine as nanoplatforms for efficient photosensitizers (PSs). Purpurin 18 (Pp18) was selected as a biosourced chlorin photosensitizer combining the efficient single oxygen generation ability and suitable absorption in the biological spectral window. The simple reaction of activated FONPs with Pp18, which contains a reactive anhydride ring, yielded nanoparticles containing both Pp18 and Cp6 derivatives. These functionalized nanoparticles combine solubility in water, high singlet oxygen generation quantum yield in aqueous media (0.72) and absorption both in the near UV region (FONPS) and in the visible region (Soret band approximately 420 nm as well as Q bands at 500 nm, 560 nm, 660 nm and 710 nm). The functionalized nanoparticles retain the blue fluorescence of FONPs when excited in the near UV region but also show deep-red or NIR fluorescence when excited in the visible absorption bands of the PSs (typically at 520 nm, 660 nm or 710 nm). Moreover, these nanoparticles behave as efficient photosensitizers inducing colorectal cancer cell (HCT116 and HT-29 cell lines) death upon illumination at 650 nm. Half maximal inhibitory concentration (IC50) values down to, respectively, 0.04 and 0.13 nmol/mL were observed showing the potential of FONPs[Cp6] for the PDT treatment of cancer. In conclusion, we have shown that these novel biocompatible nanoparticles, which can be elaborated from biosourced components, both show deep-red emission upon excitation in the red region and are able to produce singlet oxygen with high efficiency in aqueous environments. Moreover, they show high PDT efficiency on colorectal cancer cells upon excitation in the deep red region. As such, these functional organic nanoparticles hold promise both for PDT treatment and theranostics.

## 1. Introduction

Photodynamic therapy (PDT) using combinations of natural [1] or synthetic photosensitizers [2] (PSs), light and molecular oxygen has been used successfully for the clinical treatments of cancers [3,4,5] or other non-malignant diseases such as age-related macular degeneration (AMD) [6], psoriasis [7] or antibacterial chemotherapy [8] as well as for waste water treatment [9]. Because PDT allows more localized treatment with possibly lower side effects and low invasiveness, PDT represents an interesting therapeutic option to or in addition to chemotherapy. Upon excitation of the PSs at appropriate wavelengths [10], the PSs singlet excited state may deactivate to the triplet state by intersystem crossing. Effective PSs used in PDT usually have high intersystem crossing quantum yield. The PS triplet state is then able to generate reactive oxygen species (ROS) from O_2_ by two distinct mechanisms: the type I mechanism which involves electron transfer (producing superoxide anion radical O_2_^−^•, hydroxyl radical OH• and H_2_O…), or the type II mechanism which involves energy transfer (producing singlet oxygen ^1^O_2_ via triple-triplet energy transfer). Both type I and type II reactions can take place simultaneously. However, in most cases, the PDT occurs via the type II pathway [11]. Interestingly, PDT limits the cytotoxic effect to the proximate area of the PSs’ excitation since ^1^O_2_ has a very short half-life [12] in biological systems (<200 ns).

Various PSs have been used for PDT, as reviewed in the literature [3,4,13,14,15,16]. Among them, porphyrinoids [17,18] hold a peculiar place, as Photofrin^®^ and Foscan^®^ [19] were the very first compounds to be used in clinical protocols. However, most PSs are hydrophobic and tend to self-aggregate [20], which may induce a decrease of the therapeutic effect. Consequently, various methods have been developed to circumvent this limitation and increase PS bioavailability. One way is to conjugate the PSs with hydrophilic moieties. Among them, some can also target the cancer cells, including sugars [21,22,23], peptides [24,25,26] or even antibodies [27]. Another option is to modify the structure of the porphyrinic PS by adding bulky substituents or to graft the PS on nanocarriers in order to avoid aggregation [28,29,30]. Thanks to the development of nanomedicine [31], a wide range of organic or inorganic nanocarriers were used for PS delivery [32,33]. Some of them may be prepared from natural compounds [34] such as peptides [35], polysaccharides [36] or lipids [37], improving their biocompatibility.

In this context, we have decided to use specific nanocarriers, i.e., soft fluorescent organic nanoparticles (FONPs) which combine very high water-solubility, biocompatibility bright blue fluorescence properties in aqueous media and a large two-photon absorption response in the biological spectral window [38]. These nanoparticles were previously shown to internalize preferentially into cancer cells [38]. Moreover, FONPs present numerous carboxylate and amino surface groups allowing further conjugation with bioactive compounds. For instance, a highly hydrophobic cytotoxic compound (i.e., paclitaxel: PTX) was grafted onto FONPs via an ester bond yielding bioavailable prodrug systems which retain water solubility. The FONPs[PTX] nanoparticles were found to internalize into glioblastoma cells, releasing PTX within the cancer cells thanks to enzymatic processes, and subsequently inducing cell death [38]. We also showed previously that FONPs could be functionalized with the potent PS tetrakisphenylporphyrin (TPP), yielding FONPs[TPP] that internalize into cancer cells and can be imaged by two-color two-photon imaging [39]. TPP displays a high quantum yield of ^1^O_2_ generation (0.66) in organic solvents such as toluene or chloroform [40]. Yet, TPP shows poor absorption in the biological spectral window (650–900 nm) and tends to self-assemble when confined into close proximity, which is deleterious to its optical properties.

We thus decided to use a different PS and we selected chlorin derivatives. These PSs show much more intense Q bands in the red region. Among them, Chlorin p6 (Cp6) and Purpurin 18 (Pp18) are biosourced chlorin photosensitizers which have attracted a lot of interest as PSs for the PDT treatment of cancers [41,42,43,44,45,46,47,48]. Thanks to its absorption maximum located at 700 nm and its large singlet oxygen quantum yield in organic solvents, Pp18 is a very attractive PS for PDT. Yet, Pp18 is a very hydrophobic compound and its structure contains a sensitive fused anhydride. Interestingly, Cp6 which shows an absorption maximum at 665 nm can easily be obtained by opening the fused anhydride of Pp18 with a nucleophile such as an amine [49]. Along this line, previous studies have shown that polyaminated Cp6 conjugated with cellulose nanocrystals or silica nanoparticle exhibited high in vitro phototoxicity against human keratinocyte and colorectal (Colo-205) and oral (Nt8e) cancer cell lines, respectively [49,50].

As previously mentioned, Pp18 and its derivatives are hydrophobic. This is a limitation for their therapeutic use in PDT. Several teams have worked on different approaches to formulate Pp18 using water-dispersible entities as nanocarriers [48,49,50,51]. The goal of the present work was to take advantage of the properties of FONPs as nanocarriers to produce fully organic and biocompatible fluorescent nanoparticles functionalized with Pp18 and its derivative (including Cp6) as novel PDT agents. This new nano-formulation will hereafter be called FONPs[Cp6], though it includes both Pp18 and Cp6 as PSs.

FONPs[Cp6] were evaluated by electronic microscopy. The spectrophotometric and fluorescence properties were investigated in water and phosphate buffered saline solution (PBS). The pharmacological effect was determined by measuring (i) the ROS generation upon light irradiation by using Singlet Oxygen Sensor Green (SOSG) as a chemical probe and (ii) the in vitro phototoxicity against two colorectal cancer cell lines (HCT116 and HT-29).

## 2. Results and Discussion

### 2.1. Characterization of the FONPs[Cp6]

**Figure 1:**
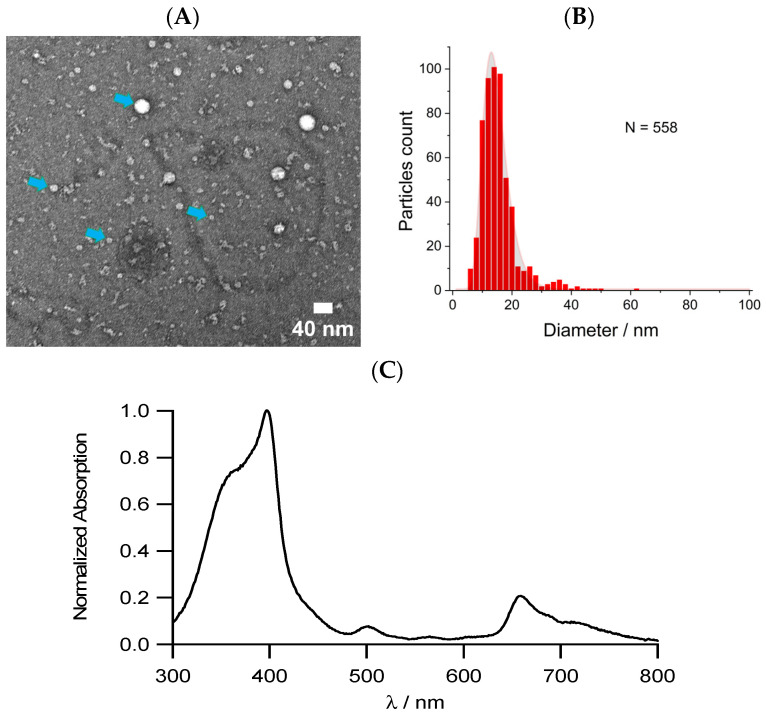
(**A**) TEM images of FONPs[Cp6], blue arrows show specific FONPs of different sizes; (**B**) size distribution of FONPs[Cp6] (TEM) fitted with a log normal; (**C**) UV-Vis absorption spectrum of FONPs[Cp6] in H_2_O.

The FONPs[Cp6] were characterized by transmission electron microscopy (TEM). As shown in Figure 1, a size distribution ranging between 10 nm and 20 nm was observed, giving a mean diameter of 12.9 nm. Small amounts of larger nanoparticles (of approximately 25 and 35 nm in diameter) are also noted (Figure 1B).

**Scheme 1:**
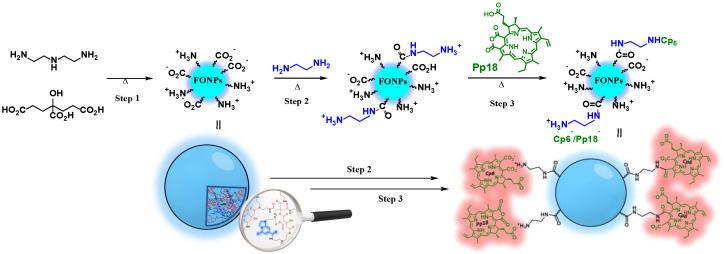
Synthetic pathway for the preparation of FONPs[Cp6]. Step 1: synthesis of the fluorescent organic nanoparticles (FONPs); step 2: activation to (FONPs^NH2^); step 3: conjugation with Pp18. The inner structure of the nanoparticle (FONPs platform) is pictured in the bottom left, showing the endogenous chromophores that are responsible for the blue fluorescence generated upon excitation in the near UV region.

Importantly, FONPs[Cp6] are hygroscopic and retain good solubility in aqueous media, thus allowing the investigation of their photophysical and photochemical properties in aqueous environments. The UV-visible absorption spectrum of the FONPs[Cp6] in water (Figure 1C) shows the presence of the endogenous chromophores of FONPs [52] (absorption maximum approximately 360 nm) and the characteristic bands of both Cp6 derivatives and Pp18 and, i.e., the intense Soret band at 400 nm and the Q bands noticeable at approximately 500 nm, 560 nm, 660 nm and 710 nm. The absorption spectra indicate that Pp18 has been grafted to FONPs^NH2^ both (i) via covalent bonds resulting from the opening of the fused anhydride of Pp18, (leading to two isomers NH-Cp6) and (ii) via ionic interaction (NH_3_^+^-CO_2_^−^) thanks to the pendant acidic function of Pp18. Indeed, both the characteristic Q band of Pp18 at approximately 700 nm and characteristic Q bands of Cp6 and Pp18 are observed, with some overlaps. A broadening is also observed due to the presence of the two isomers resulting from the reaction of the amino surface function of the FONPs on the fused anhydride cycle on the two different positions (see Section 3.1 and Scheme 1). From the Q bands’ absorbance of stock solutions of FONPs[Cp6], we derived estimated values of the loading of Cp6 (~30 μmol·g^−1^) and Pp18 (~6 μmol·g^−1^) for the dry nanoparticles (hereafter, represented as FONPs[Cp6]). Keeping in mind that Pp18 units are expected to slowly hydrolyze to Cp6 derivatives in aqueous environments; the amount of total loading of PSs is evaluated to approximately 35 μmol·g^−1^.

### 2.2. Fluorescence Properties

**Figure 2:**
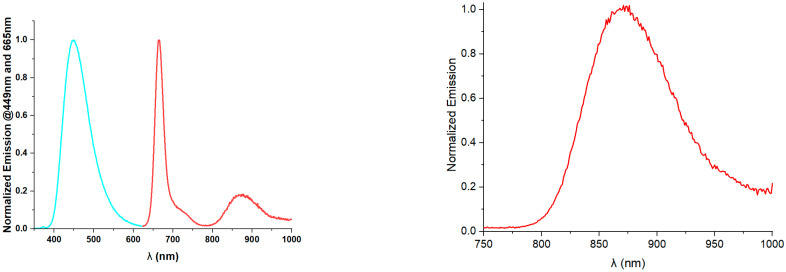

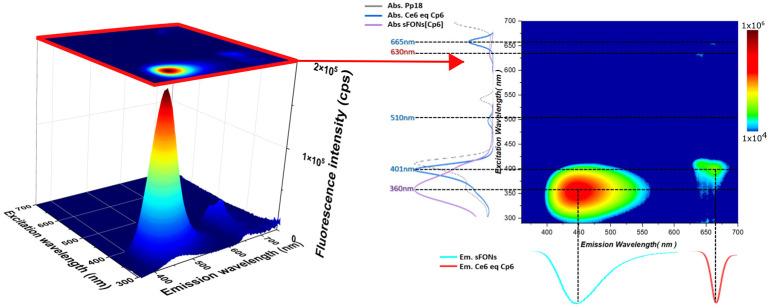
(**Top**) Emission spectra of FONPs[Cp6] in PBS under excitation at 330 nm (blue) and 520 nm (red) (**left**). Emission spectra of FONPs[Cp6] in PBS under excitation at 710 nm (**right**). (**Bottom**) Two-dimensional fluorescence projection (**right**) from 3D fluorescence experiment (**left**).

Thanks to their high water-solubility, the fluorescence properties of the FONPs[Cp6] could also be investigated in aqueous media (distillated water and PBS). The fluorescence emission spectra recorded in PBS are shown in Figure 2.

As observed from Figure 2, the FONPs[Cp6] retain both the blue emission (maximum at 448 nm) of the FONPs’ endogenous chromophores when excited in the UV region (330 nm where the endogenous chromophores are more selectively excited) and the deep-red emission (665 nm, 720 nm) upon excitation in the green visible (520 nm), originating from the immobilized Cp6 derivatives. In addition, a very weak NIR emission is observed in between 820 nm and 950 nm (peaking at 880 nm) upon excitation at 710 nm. This originates from Pp18 and thus expected to be quite low due to the low amount of remaining (non-hydrolyzed) Pp18 and the very low fluorescence quantum yield of NIR emission, especially in water. In contrast, the emission quantum yield of the blue emission remains significant (20%), though lower than that of non-conjugated FONPs^NH2^ (30%), indicating that competing deactivation processes occur in the excited state of the endogenous chromophores in the presence of the PSs. These could be due to either energy transfer (the blue emission of the endogenous overlapping with the intense Soret band of Cp6 and Pp18 derivatives) or to fast photoinduced electron transfer followed by charge recombination, thereby bypassing fluorescence emission. Three-dimensional fluorescence experiments show that Förster resonance energy transfer (FRET) may indeed be involved (Figure 2, bottom right: green spot corresponding to λ_exc_ = 300 nm and λ_em_ = 665 nm). Yet, this transfer is not quantitative as two-thirds of the blue emission is retained upon excitation at 330 nm (Table 1).

The deep-red emission fluorescence quantum yield of Cp6 (excitation at 520 nm) is comparatively much lower (4–9%), in agreement with the band gap law, but remains acceptable, especially in water and aqueous environments. These fluorescence properties are of particular interest for monitoring the internalization of these nanocarriers loaded with PSs into cells while the NIR emission of Pp18 could be of interest for in vivo imaging.

### 2.3. Singlet Oxygen Generation

**Figure 3:**
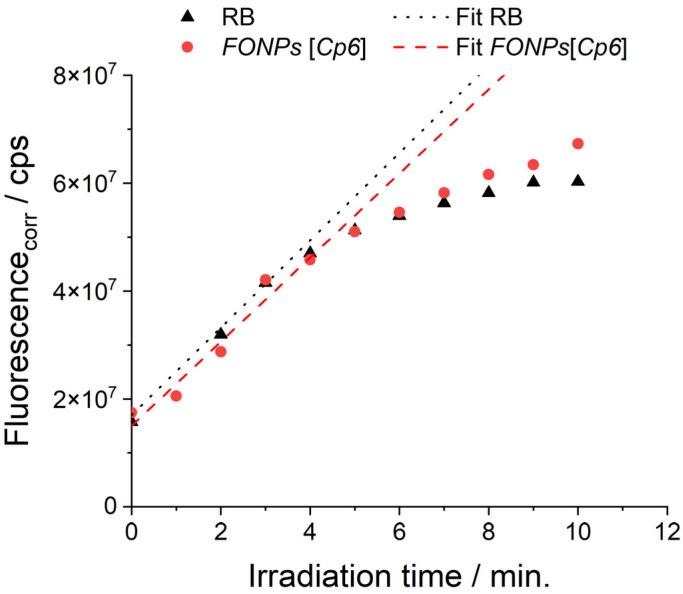
Kinetic of oxidation of SOSG probe in PBS in the presence of Rose Bengal with 530 nm (25 mW) or FONPs[Cp6] upon 405 nm (25 mW) illumination.

To investigate the PDT performance of FONPs[Cp6], we first measured its ROS generation upon excitation by using Singlet Oxygen Sensor Green (SOSG) as a chemical probe. SOSG has been used in a range of biological systems that are known to generate singlet oxygen [53]. The excitation was carried out with a 405 nm LED (Soret band of the PSs). Fluorescence spectra of the solution were recorded regularly to monitor the variation of the fluorescence intensity. As shown in Figure 3, the fluorescence intensity of SOSG at 534 nm increased significantly over time after incorporating 4 µL of SOSG (0.5 mM in MeOH) into 2.8 mL of an aqueous solution of FONPs[Cp6] upon excitation at 405 nm. In a control experiment involving SOSG without FONPs[Cp6], only a faint change was observed. These results indicate a fast generation of ^1^O_2_ from FONPs[Cp6].

The singlet oxygen generation quantum yield (ϕ^Δ^) of FONPs[Cp6] was determined in PBS using a standard rose bengal (RB) whose ϕ^Δ^*_Ref_* value is 0.76 in PBS [54]. The ^1^O_2_ generation during the illumination of FONPs[Cp6] or RB was monitored by the fluorescence of the oxidized form of the SOSG probe. As the absorption coefficient values of SOSG in its oxidized and reduced form are slightly different, for each time point (*t*), fluorescence intensity (∫fluorescence) was corrected by the measured absorbance at 504 nm using Equation (1):

(1) $${Fluorescence}_{corr}(t)=\frac{\int fluorescence}{1-10^{Abs504nm(t)}}$$

Then, ^1^O_2_ quantum yield (ϕ^Δ^) can be estimated using Equation (2):

(2) $$\phi^{\Delta }=\phi^{\Delta_{Ref}}\cdot \;\frac{k^{SOSG}\;}{k^{{SOSG}_{Ref}}}$$

where *k^SOSG^* and *k^SOSG^_Ref_* are the first order reaction rate constants of generation of the oxidized form of SOSG in the presence of FONPs[Cp6] or RB, respectively, (ϕ^Δ^*_Ref_* = 0.76).

From the measurements in the linear part of the curve (Figure 3), we derived the slope values *k^SOSG^* = 7.8 × 10^6^ min^−1^ and *k^SOSG^_Ref_* = 8.1 × 10^6^ min^−1^, from which we could derive a ϕ^Δ^value of 0.72 for FONPs[Cp6] in PBS. Hence, the FONPs nanoparticles behave as smart nanocarriers, providing water solubility to the hydrophobic photosensitizers as well as excellent singlet oxygen generation quantum yield in aqueous environments.

### 2.4. In Vitro Photoirradiation Studies of FONPs[Cp6] on Colorectal Cancer Cells

**Figure 4:**
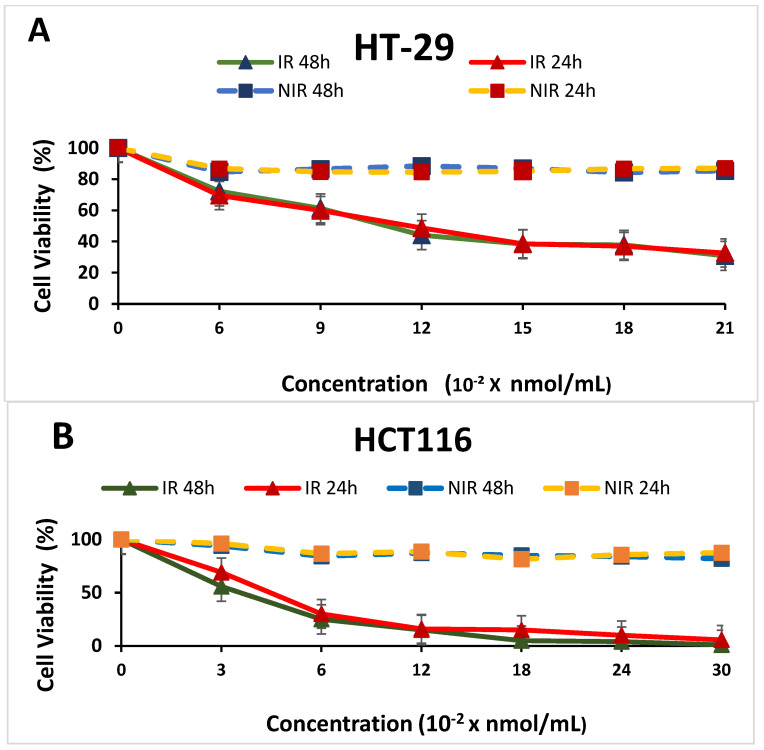
In vitro phototoxic effect of FONPs[Cp6] on HT-29 (**A**) and HCT116 (**B**) cell lines. Data are shown as mean ± SEM (*n* = 3). Illumination conditions: 38 mW/cm^2^ during 32 min.

The phototoxic effect of FONPs[Cp6] in vitro was examined on two human colorectal cancer cell lines: HT-29 and HCT116. For this purpose, we treated these two cell lines with increasing concentrations of FONPs[Cp6], then cells were irradiated (IR) or not (NIR) with red illumination (650 nm). The cytotoxic effects were investigated by MTT assay. Our results revealed that FONPs[Cp6] did not induce any significant toxicity in the dark on either HCT116 or HT-29 cell lines. In contrast, after PDT irradiation, the cell viability decreases rapidly in a dose-dependent manner in both cell lines (Figure 4).

HT-29 cells were or were not seeded in 96-well culture plates for 24 h in DMEM medium before treatment with different concentrations of FONPs[Cp6]. After 24 h incubation, these cells were or were not irradiated with a 650 nm lamp (75 J/cm^2^). Cell viability was monitored by MTT assay 24 h and 48 h after irradiation, as a percentage of each condition compared to untreated ones. Data are shown as mean ± SEM (*n* = 3).

HCT116 cells were or were not seeded in 96-well culture plates for 24 h in RPMI medium before treatment with different concentrations of FONPs[Cp6]. After 24 h incubation, these cells were or were not irradiated with a 650 nm lamp (75 J/cm^2^). Cell viability was determined by MTT assay 24 h and 48 h after irradiation, as a percentage of each condition compared to untreated ones. Data are shown as mean ± SEM (*n* = 3).

Our results showed that 48 h after irradiation, the HCT116 cells are more sensitive to treatment with FONPs[Cp6] than the HT-29 cells with IC50s of 0.04 nmole/mL (corresponding to 1.14 µg of Cp6/mL) and 0.13 nmole/mL (corresponding to 3.62 µg of Cp6/mL) respectively, (Table 2).The higher efficacy of photosensitizers after illumination against HT116 versus HT-29 colorectal cancer cell lines is well known and is in agreement with the literature [47].

These results demonstrate that FONPs[Cp6] are potentially active PSs for PDT. In comparison with the literature, FONPs[Cp6] showed a better efficacy than nanoparticles functionalized with porphyrinic PSs. As an example, in the same conditions and after PDT treatment of the same colorectal cancer cell lines, Bouramtane et al. showed that silica-xylan nanoparticles bearing porphyrin (TTPOH) as a photosensitizer display cytotoxic activity, with IC50 values of 0.0726 and 0.550 nmol/mL after 48 h against HCT116 and HT-29 cancer cell lines, respectively [55]. With pheophorbide-a covalent linked to xylan nanoparticles, in vitro phototoxicity (IC50 values) was 810 nmol/mL after 48 h against the HT-29 cell line [56]. Hence, the FONPs[Cp6] represent promising biosourced and bioinspired material for the PDT treatment of colorectal cancer. In that perspective, in vivo tests will be conducted in the near future.

## 3. Materials and Methods

### 3.1. Synthesis

The starting materials were purchased from Sigma-Aldrich Chimie (Saint Quentin Fallavier, France), TCI EUROPE N.V. (Haven, Belgium) and Thermo Scientific Chemicals (Karlsruhe, Germany). Spirulina maxima was purchased from the Claudine Vallée EURL (Chanzeux, France).

The synthesis of FONPs[Cp6] was achieved in three steps (Scheme 1). FONPs were obtained from the controlled polycondensation of citric acid with diethylenetriamine (DETA): step 1. A different protocol was elaborated in comparison to the standard protocol reported earlier [38,39]. Our motivation was to implement a more robust and easily scalable protocol. The ammonium citrate salt was first prepared by mixing equimolar amounts of DETA and citric triacid in pure water, then evaporating the water under vacuum to obtain a dry solid (i.e., diethylenetriamonium citrate). The salt was subsequently heated at 200 °C for 30 min leading to a brownish-yellowish crude material. This crude material was washed with ethanol then dried under vacuum leading to a yellowish solid (bare FONPs). Bare FONPs were then enriched in NH_2_ surface groups by heating them in ethylene diamine at 120 °C for 16 h (Scheme 1, step 2), leading to FONPs^NH2^ [39].

In parallel, Pp18 was prepared from cyanobacterium *Spirulina maxima* as previously described [57]. After acetone extraction, the chlorophyll *a* was treated with a NaOH solution in the presence of oxygen to enable the saponification of the ester groups and the formation of the six-membered cyclic anhydride. The subsequent acidic treatment furnished Pp18.

Experimental protocol: Pigments are extracted twice from *Spirulina maxima* in dry powder form (50 g) with acetone (2 × 1 L) by stirring at 60 °C (reflux) for 2 × 1 h. The dark green extract is filtered and the filtrate (2 L) is reduced to 1 L by partial evaporation under vacuum using a rotary evaporator. A 6M NaOH solution (200 mL) is added and air is bubbled through the mixture which is stirred vigorously for 2 h. Concentrated hydrochloric acid (100 mL) is then added and the resulting mixture is stirred at room temperature for 30 min. The solution is decanted. The organic layer is dried over MgSO_4_ and evaporated under reduced pressure. The residue is purified using silica gel flash column chromatography (CH_2_Cl_2_/MeOH = 92/8) to afford Purpurin 18 (241.3 mg). ^1^H-NMR (500 MHz, CDCl_3_, TMS): δ_ppm_ = 9.55 (s, 1H, 10-H), 9.34 (s, 1H, 5-H), 8.56 (s, 1H, 20-H); 7.86 (dd, *J =* 17.8, 11.6 Hz, 1H, 3^1^-CH); 6.28 (d, *J =* 17.8, 1H, trans-3^2^-CH_2_); 6.17 (d, *J =* 11.6, 1H, cis-3^2^-CH_2_); 5.18 (dd, *J =* 9.3, 1.8 Hz, 1H, 17H); 4.38 (q, *J =* 7.3 Hz, 1H, 18-H); 3.74 (s, 3H, 12-CH_3_); 3.61 (q, *J =* 7.7 Hz, 2H, 8^1^-CH_2_); 3.33 (s, 3H, 2-CH_3_), 3.14 (s, 3H, 7-CH_3_); 2.76 (m, 1H, 17^2^-H), 2.53–2.42 (m, 2H, 17^1^-H), 1.98 (m, 1H, 17^2^-H); 1.73 (d, *J =* 7.3 Hz, 3H, 18-CH_3_); 1.65 (t, *J =* 7.7 Hz, 3H, 8-CH_3_); 0.19 (brs, 1H, NH); −0.10 (brs, 1H, NH).

Finally, FONPs^NH2^ were reacted with Pp18 (Scheme 1, step 3) in a biphasic medium (H_2_O/toluene) under vigorous stirring and heating at 100 °C. During this step, the FONPs^NH2^ surface NH_2_ groups may react with the exocyclic anhydride of Pp18 leading to the covalent grafting of chlorin *p6* (Cp6) via an amide bond. In parallel, Pp18 may slowly hydrolyze leading to Cp6. Both Cp6 and Pp18 have carboxylic functions which can also be extracted in the water phase by interaction with the polyaminated FONPs^NH2^_._ While the FONPs^NH2^ are soluble in the aqueous phase, Pp18 is soluble in toluene. During the reaction, the colored toluene phase gradually fades while the aqueous solution turns red.

Experimental protocol: To a solution of 350 mg of FONPs^NH2^ in 3 mL of distilled water, were added 35 mL of toluene, then 14 mg of Pp18 (25 µmoles). The reaction mixture was stirred vigorously for 90 min while heating at 100 °C in the dark under argon. Stirring was continued overnight under an inert atmosphere. The toluene phase, which became colorless, was then decanted and discarded. The water phase was washed with toluene then freeze-dried. A final washing with dichloromethane was carried out: 310 mg of dark-green solid was obtained and stored in a freezer at −18 °C.

### 3.2. Characterization

Transmission electron microscopy (TEM): The sizes of FONPs-[PS] were determined by TEM imaging which was performed on a HITACHI H7650 (Krefeld, Germany) at 80 KV. Copper grids coated with a carbon membrane were pre-treated using the glow discharge technique. One droplet of the aqueous nanoparticle solution was deposited on the grid; the excess liquid was gently drawn-off with paper and the sample was further stained with uranyl acetate. The nanoparticles were randomly and manually counted using the ImageJ program (using a circle selection). The diameter of each nanoparticle was measured and the results were given as a medium size (diameter) of the overall counted nanoparticles. For the statistics, 558 nanoparticles were counted.

UV-visible absorption and fluorescence spectroscopies: All photophysical properties were investigated with freshly prepared air equilibrated solutions at room temperature (293 K). UV/Vis absorption spectra were recorded using a Jasco V-570 spectrophotometer (Lisses, France). Steady-state fluorescence measurements were performed on diluted solutions (optical density < 0.1) contained in standard 1 cm quartz cuvettes using a Horiba FluoroMax spectrometer (Palaiseau, France) or a Horiba Fluorolog spectrometer (Palaiseau, France) in photon-counting mode. Fully corrected emission spectra were obtained for each compound at λ_ex_ = λ_abs_^max^ with an optical density at λ_ex_ ≤ 0.1 to minimize internal absorption.

### 3.3. Singlet Oxygen Detection

To evaluate the singlet oxygen generation (SOG) quantum yield of FONPs[Cp6] (1.3 μM), Singlet Oxygen Sensor Green (SOSG) was added (4 µL of a 5 mM solution in MeOH) to the solution of FONPs[Cp6] in PBS (2500 µL, 1.3 μM) in a quartz cuvette and irradiated by a 240 mW 405 nm LED at a distance of 11 cm, with up to 30 min of total illumination time. Fluorescence spectra of the solution were recorded each minute for the first 10 min, then every 5 min after. The control experiment was performed by irradiating the SOSG solution in the same conditions in the absence of FONPs[Cp6]. The SOSG fluorescence was red out at 534 nm after the irradiation at 405 nm to determine the samples’ SOG. The samples’ SOG was evaluated using the SOSG fluorescence enhancement and compared with the background of control sample.

### 3.4. In Vitro Photo-Irradiation Studies

#### 3.4.1. Cell Lines

The two human colorectal cancer cell lines HT-29 and HCT116 used were purchased from the American Type Culture Collection (ATCC-LGC Standards, Mosheim, France). HT-29 cells were grown in the DMEM medium while HCT116 were in the RPMI medium. Culture media was completed with 10% fetal bovine serum (FBS), 1% L-glutamine and 100 U/mL penicillin and 100 μg/mL streptomycin [all reagents are from Gibco BRL (Cergy-Pontoise, France)]. The cultures were placed at 37 °C in a humidified incubator with 5% CO_2_.

#### 3.4.2. FONPs[Cp6] Phototoxicity

The FONPs[Cp6] stock solution was prepared by dissolving 10 mg in 3 mL dimethyl sulfoxide (DMSO). The final concentrations were obtained by dilution in an appropriate culture medium for each cell line, the final percentage of DMSO in each condition being less than 0.1% in all cases. The PDT protocol proceeded for all experiments as follows: HT-29 cells were seeded at 2.1 × 10^4^, and HCT116 cells at 1.2 × 10^4^ cells/cm^2^. After 24 h, the cells were treated or not with FONPs[Cp6]. After 24 h post-treatment, the medium was replaced with a red phenol-free medium before being irradiated with a 650 nm lamp (Photocure and Cosmedico) as a light source at 75 J/cm^2^ (38 mW/cm^2^ during 32 min). The cell viability was then measured 24 h and 48 h after irradiation by normalizing to untreated conditions. The phototoxicity was examined using the 3-(4,5-dimethylthiazol-2-yl)-2,5- diphenyltetrazolium bromide (MTT) assays.

## 4. Conclusions

The biocompatible fully organic soft FONPs are of great interest for solubilizing the hydrophobic biosourced photosensitizers Pp18/Cp6 in aqueous environments. Moreover, the resulting nanoparticles retain both the deep-red fluorescence of the photosensitizers in aqueous solutions and show an impressive singlet oxygen generation quantum yield in water (0.72). In addition, the present work showed that such formulation of PSs, while maintaining their photochemical properties, is effective as PDT agent against two types of colorectal cancer cells (HT-29 and HCT116) by producing 72% of singlet oxygen. In addition, thanks to its deep-red NIR fluorescence, this nanoformulation offers promises for theranostics and guided therapy. Based on these positive results, additional in vivo tests will be conducted on small animals.

## Figures and Tables

**Table 1:** Fluorescence properties of FONPs^NH2^ and FONPs[Cp6] in water.

Compound	λ_abs_^max^ (nm)	λ_exc_ (nm)	λ_em_^max^ (nm)	Φ_f_ ^a^
FONPs^NH2^	360	360	450	0.30 ^b^
FONPs[Cp6]	360, 400, 500 (sh), 550 (sh), 665, 710	330	448	0.20 ^b^
		520	665, 720	0.04 ^c^, 0.09 ^d^
		710	880	n.d.

^a^ Experimental fluorescence quantum yield values determined using; ^b^ quinine bisulfate in 1NH_2_SO_4_ (Φ_f_ = 0.546); ^c^ 4-(dicyanomethylene)-2-methyl-6-(4-dimethylaminostyryl)-4H-pyran (DCM) in ethanol (Φ_f_ = 0.437) and ^d^ indocyanine green in dimethylsulfoxide (Φ_f_ = 0.11) as standards.

**Table 2:** IC50 (for FONPs[Cp6] in µg/mL and for active PSs in nmole/mL) values determined with MTT assays on HCT116 and HT-29 cell lines.

FONPs[Cp6]	IC50/IR 24 h		IC50/IR 48 h	
HCT116	1.40 µg/mL	0.05 nmol/mL	1.14 µg/mL	0.04 nmol/mL
HT-29	3.86 µg/mL	0.15 nmol/mL	3.62 µg/mL	0.13 nmol/mL

## Data Availability

Data are contained within the article.
